# Molecular analysis of Shiga toxin-producing *Escherichia coli* O157:H7 and non-O157 strains isolated from calves

**DOI:** 10.4102/ojvr.v85i1.1621

**Published:** 2018-10-17

**Authors:** Maryam Kohansal, Ali Ghanbari Asad

**Affiliations:** 1 Department of Medical Biotechnology, Fasa University of Medical Science, Iran; 2 Department of Biology, Payame Noor University (PNU), Iran

## Abstract

Shiga toxin-producing *Escherichia coli* (STEC) O157 and non-O157 are food-borne pathogens and contaminants of foods of animal origin. This study was conducted to investigate the presence of virulence and integrase genes in STEC isolates from diarrhoeic calves in Fars Province, Iran. Five hundred and forty diarrheic neonatal calves were randomly selected for sampling. Rectal swabs were collected and cultured for isolation and identification of *E. coli* following standard methods. The isolates were analysed for the presence of class 1 integrons and bacterial virulence factors using polymerase chain reaction (PCR). Antimicrobial susceptibility testing was performed using the Kirby–Bauer disc diffusion method. Out of 540 diarrhoeic faecal samples, 312 (57.7%) harboured *E. coli* and 71 (22.7%) of them were identified as STEC: 41(69.5%) carried the *stx2* gene, 21 (35.6%) carried the *stx1* gene and 3 (5%) carried both. Twenty-six (44%) of the isolates showed the *eae* gene. Among the STEC isolates examined for susceptibility to eight antimicrobial agents, erythromycin and penicillin (96.8%) resistance were most commonly observed, followed by resistances to ampicillin (71.8%), tetracycline (62.5%) and trimethoprim/sulfamethoxazole (39%). Integrons were detected by PCR in 36% of the STEC tested isolates, 57 (89%) of which showed resistance to at least three antimicrobial agents. Our findings should raise awareness about antibiotic resistance in diarrhoeic calves in Fars Province, Iran. Class 1 integrons facilitate the emergence and dissemination of multidrug-resistance (MDR) among STEC strains recovered from food animals.

## Introduction

New research provides the strongest evidence that Shiga toxin-producing *Escherichia coli* (STEC) non-O157:H7 and in particular serogroup O157 are linked to severe gastrointestinal diseases (Dehkordi et al. 2014). The clinical manifestations of STEC infection can vary, from asymptomatic carriage to very serious illnesses such as haemolytic uremic syndrome (HUS), thrombocytopenic purpura (TTP) and haemorrhagic colitis (HC) (Thomas et al. 2012). Estimates vary, but experts suggest that gastrointestinal infections are responsible for approximately 1.5 million deaths per year, over 90% of which are in developing countries (Montenegro et al. 2011). For instance, non-O157:H7 serogroups are found in more than 36 000 cases of infections annually and at least 73 000 are infected with O157:H7 serogroup in the United States (US) (Zhao et al. 2001).

Studies have revealed that O157 and non-O157 strains of cattle origin can cause the disease in humans via consumption of raw milk and undercooked meat. In fact, cattle, especially young animals, are known to be the primary reservoirs of both non-O157 and O157 STEC (Moura et al. 2012). The pathogenicity of STEC is associated with Shiga toxin (stx) encoded by Shiga toxinogenic (*stx*) genes 1, 2 (*stx1* and *stx2*) and an outer membrane protein which is encoded by the chromosomal *eae* gene (Pradel et al. 2008).

The problems with some new STEC strains isolated from neonatal calf diarrhoea (NCD) (Rigobelo et al. 2008), which is recognised as a disease complex characterised by acute, undifferentiated diarrhoea in newborn calves, are that antibiotic multiresistance (De Verdier et. al. 2012) and STEC strains can be transmitted to humans by contact occupational exposure and the food chain (Schroeder et al. 2002). Epidemiological observations show high levels of antimicrobial resistance in bacterial pathogens from veterinary and human medicine (Zhao et al. 2001). This has led to the discovery that these bacteria are able to acquire antibiotic resistance by resistance-conferring genes, many of which are carried on transposons, plasmids or integrons (Bakhshi, Najibi & Sepehri-Seresht 2014). An integron is mainly composed of an integrase gene that encodes a site-specific recombinase, by which an insertion site of integron is recognised. Moreover, an integron contains a variable region which is the place for gene cassettes to be inserted (White et al. 2001). Depending on the sequence of the encoded integrases (*intI*) catalysing excision and integration of deoxyribonucleic acid (DNA) units, eight distinct integron classes have been identified up to now, and class 1 integrons have shown to be the major contributors to multidrug-resistant (MDR) infections in the Enterobacteriaceae family (Singh et al. 2005).

Many studies in various countries including Iran have shown that the distribution of integrons among enteric bacteria has increased over time (Eftekhari et al. 2013; Gonzalez et al. 1998; Hamada, Oshima & Tsuji 2003; Martinez-Freijo et al. 1998, 1999; Najibi et al. 2012). In Iran, only a few studies have reported antimicrobial resistance properties and virulence genes in the pathogenic *E. coli* (Bakhshi et al. 2014; Shahrani et al. 2014). Unfortunately, there is no conclusive data on the distribution of virulence genes and the antimicrobial resistance properties of STEC strains isolated from Iran, particularly from Fars, which is one of the major agricultural and animal husbandry areas in Iran, with nearly 400 000 cattle and 8 000 000 sheep and goats (Shams et al. 2012).

## Materials and methods

### Study design and study areas

#### Sampling and *Escherichia coli* identification

A total of 540 recto-anal mucosal swabs from diarrhoeic calves (< 30 days of age) were collected over 1 year from November 2015 to November 2016.

These calves were raised on 33 farms from eight geographic areas in Fars Province, including industrial, semi-industrial and traditional farms, with a herd size of 25–500 cows. These farms had a recognised scouring problem in neonatal calves. Sick calves which showed abnormal faecal consistency and/or signs of dehydration and weakness were selected. None of them had been vaccinated. All samples were immediately placed in cooled boxes and transported to the laboratory. The swab samples were incubated overnight at 37 *°*C in trypticase soy broth (TSB) (Merck KgaA, Darmstadt, Germany). Each sample was then streaked onto MacConkey’s agar (MC, Merck, Germany) (24 hours at 37 °C). Lactose positive colonies were cultured on eosin methylene blue agars (EMB, Merck, Germany) (24 h at 37 °C). Green colonies with a metallic lustre were considered typical *E. coli* colonies. Such colonies were confirmed as *E. coli* using standard biochemical tests (citrate utilisation, indole production, glucose, lactose fermentation, urease negative and hydrogen sulphate production). The biochemically confirmed *E. coli* colonies were subjected to DNA analysis.

#### Antimicrobial susceptibility and multidrug resistance

Antimicrobial susceptibility testing against eight antimicrobials was performed on 52 O157 and 12 non-O157 STEC isolates using the disk diffusion method on Mueller Hinton agar plates (Merck, Germany) based on the Clinical and Laboratory Standards Institute (CLSI) guidelines (Wayne 2012a). The following antibiotics (PadtanTeb, Iran) were applied: chloramphenicol (C: 30 *µ*g), erythromycin (E: 25 *µ*g), ampicillin (AM: 10 *µ*g), trimethoprim/sulfamethoxazole (SXT: 30 *µ*g), penicillin (P: 10 *µ*g), enrofloxacin (ENR: 10 *µ*g), cefixime (CFM: 5 *µ*g) and tetracycline (TET: 30 *µ*g). The zone diameters were measured (to the nearest millimetre) and interpreted as intermediate (I), susceptible (S) or resistant (R) according to CLSI protocol (Wayne 2012a); intermediate strains were considered susceptible. Based on the definition proposed by an international expert, the MDR phenotype was resistant to three or more antimicrobial classes (Magiorakos et al. 2012). *E. coli, ATCC 25922* (sensitive to all these drugs), recommended by CLSI, was used as a quality control. The specified range of quality control result was published in M100-S22 (Wayne 2012b).

#### DNA extraction

A single colony of overnight TSB culture was suspended in 100 *µ*L of distilled water and exposed to boiling for 10 min at 100 °C. After a 13 min freeze, the frozen cell pellets were centrifuged at 14 000 rpm for 10 min (Dehkordi et al. 2014) and the supernatant, containing bacterial DNA, was subjected to PCR analysis.

#### Polymerase chain reaction detection of virulence factors and class 1 integron in Shiga toxin-producing *Escherichia coli* strains

Polymerase chain reaction assays were used to detect the presence of the following virulence genes coding regions including *stx1, stx2* and *eae*. To detect class 1 integron in confirmed STEC isolates, a PCR protocol was employed. Preparation of the DNA samples was done as described in previously published paper (Dehkordi et al. 2014). Primer sequences, sizes of PCR products and PCR conditions are shown in Table 1. DNA from *E. coli* O157:H7 EDL933 strain and *ATCC 25922* strains were used as positive and negative controls, respectively. The amplified DNA products were separated by 1.5% agarose gel electrophoresis (Sigma-Aldrich, St. Louis, MO, United States). The gels were stained with ethidium bromide (Merek, Germany). Visualisation of amplified products was done by ultraviolet (UV) illumination and photographed using a Kodak camera system (Gel Logic 200).

**TABLE 1:** Primers and polymerase chain reaction conditions used in this study.

Gene	Primer sequence	Size of product (bp)	PCR programme	PCR volume (25 *μ*L)	Reference
*stx1*	F: CTT CGG TAT CCT ATT CCC GG R: GGA TGC ATC TCT GGT CAT TG	484	25 cycles of 30 s at 94 °C 45 s at 50 °C 90 s at 70 °C 10 min at 70 °C	2.5 *μ*L PCR buffer 10X 1.25 *μ*L MgCl_2_ 0.5 *μ*LdNTP 1 *μ*L of each primers F & R 0.25 *μ*LTaq DNA polymerase 1 *μ*L DNA template	Tahamtan et al. (2010)
*stx2*	F: CCA TGA CAA CGG ACA GCA GTT R: CCT GTC AAC TGA GCA GCA CTT TG	779	25 cycles of 30 s at 94 °C 45 s at 50 °C 90 s at 70 °C 10 min at 70 °C	2.5 *μ*L PCR buffer 10X 1.25 *μ*L MgCl_2_ 0.5 *μ*LdNTP 1 *μ*L of each primers F & R 0.25 *μ*LTaq DNA polymerase 1 *μ*L DNA template	Tahamtan et al. (2010)
*eae*	F: AAG CGA CTG AGG TCA CT R: ACG CTG CTC ACT AGA TGT	384	25 cycles of 30 s at 94 °C 45 s at 50 °C 90 s at 70 °C 10 min at 70 °C	2.5 *μ*L PCR buffer 10X 1.25 *μ*L MgCl_2_ 0.5 *μ*LdNTP 1 *μ*L of each primers F & R 0.25 *μ*LTaq DNA polymerase 1 *μ*L DNA template	Vasconcellos et al. (2012)
*IntI*	F: TGCGGGTYAARGATBTKGATTT* R: CARCACATGCGTRTARAT	491	30 s at 94 °C, 35 s at 57 °C 25 cycles of 1 min at 70 °C 10 min at 72 °C	2.5 *μ*L PCR buffer X 10 1.25 *μ*L MgCl_2_ 1 *μ*LdNTP 1 *μ*L of each primers F & R 0.25 *μ*LTaq DNA polymerase 1 *μ*l DNA template	Tahamtan et al. (2014)

Note: B = C or G or T; K = G or T; R = A or G; Y = C or T*.

PCR, polymerase chain reaction; DNA, deoxyribonucleic acid; Taq, thermus aquaticus; *stx*, isolates carrying *stx1* and/or *stx2* genes; *eae*, isolates carrying *eae* gene; pb, base pair; *IntI*, encoded integrases.

#### Statistical analysis

The chi-square (χ^2^) test and Fisher’s exact test were used to assess whether integron-positive strains were significantly more resistant than integron-negative strains for each of the tested antibiotics. A *p* value < 0.05 was considered statistically significant. Statistical calculations were made using GraphPad Prism for Windows version 5 (GraphPad Software, San Diego, CA).

## Results

### Isolation and characterisation of Shiga toxin-producing *Escherichia coli* in calves

From 540 diarrhoeic calves, 312 samples (57.7%) were positive for *E. coli*. Shiga toxin–producing *Escherichia coli* strains were isolated from 71 (22.7%) out of the 312 samples, which possess *stx1* and/or *stx2*. Twelve (3.57%) isolates were classified as *E. coli* O157:H7 and 59 (31.19%) as non-O157.

### Characterisation of virulence genes

**FIGURE 1:**
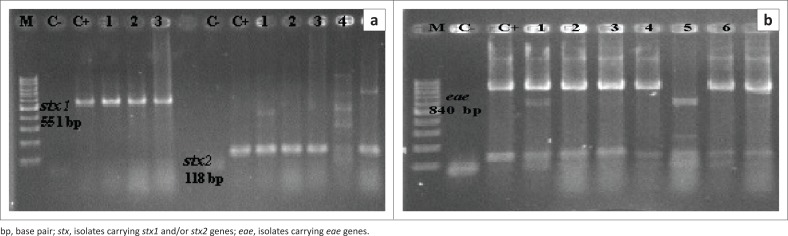
Agarose gels electerophoresis of Shiga toxin-producing *Escherichia coli* isolates. (a) Polymerase chain reaction amplification of the *stx1* (551 bp) and *stx2* (118 bp) genes. Lanes 1–3, *stx1*; lanes 1–3 and 5, *stx2* and (b) polymerase chain reaction amplification of the *eae* gene (840 bp) (lanes 1–4, 6 and 7). Lane M, 100 bp molecular size markers; Lanes C- and C+, negative and positive control.

**FIGURE 2:**
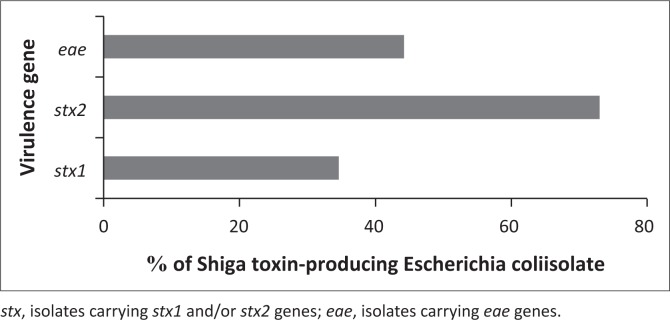
Frequency of occurrence of tested virulence genes in 71 STEC strains.

Of 312 *E. coli* strain*s*, 71 isolates (22.7%) were identified as STEC. The virulence genes *stx2, stx1* and *eae* were detected at 76%, 46.4% and 53.5% in STEC isolates, respectively. Of isolates that were not characterised as STEC, 101 (32.3%) were positive for *eae* gene (Figures 1 and 2). These findings are summarised in Table 2. Out of 59 non-O157 strains (PNU6, PNU11, PNU12 and PNU16) in the diarrhoeic calves, four were positive for both the *stx1* and *stx2* genes and three non-O157 harboured all of the *stx1, stx2* and *eae* genes.

**TABLE 2:** Distribution of virulence genes in Shiga toxin-producing *Escherichia coli* strains.

Pathotype	Serogroup	Positive sample		Number of isolates carrying specific genes													
		No.	%	*stx1*		*stx2*		*eae*		*stx2/stx1*		*stx1/eae*		*stx2/eae*		*stx1/stx2/eae*	
				No.	%	No.	%	No.	%	No.	%	No.	%	No.	%	No.	%
STEC	Non-O157	59	100.0	21	36	41	70	26	44.0	3	5.0	10	16.9	13	22.0	3	5.0
	O157	12	-	12	-	12	-	12	-	0	-	0	-	0	-	12	-
Total STEC		71	100.0	33	46	53	75	38	54	3	4.2	10	14.0	13	18.3	15	21.1
Non-STEC		241	-	-	-	-	-	63	-	-	-	-	-	-	-	-	-
Total		312	100.0	33	11	53	17.0	101	32	3	0.9	10	3.2	13	4.1	15	4.8

Overall: STEC, 71 (22.7%); Non-STEC, 241 (77.3%).

Overall: Non-O157, 52 (16.6%); O157, 12 (3.8%).

STEC, Shiga toxin-producing *Escherichia coli*; No., number; *stx*, isolates carrying *stx1* and/or *stx2* genes; *eae*, isolates carrying *eae* gene.

Note: The *eae* gene produces a 94-kDa outer membrane protein called intimin.

### Antibiotic susceptibility

**FIGURE 3:**
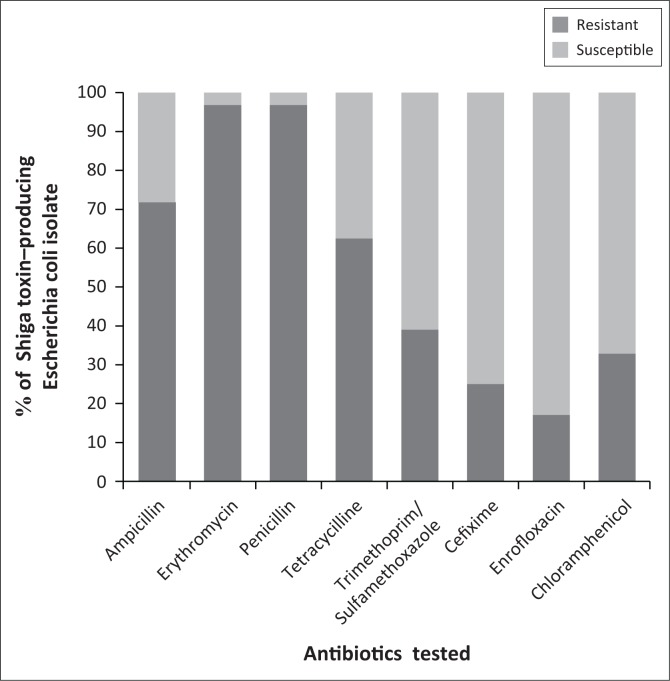
Antimicrobial susceptibility patterns in 71 Shiga toxin-producing *Escherichia coli* strains.

The antimicrobial susceptibility of 52 non-O157 and 12 O157 STEC isolates was determined by the disk diffusion method. The resistance patterns of the *E. coli* O157 strains were to penicillin and ampicillin (91% – 8%), followed by tetracycline, erythromycin and cefixime (66% – 25%). Nine (75%) of the 12 O157 strains exhibited multidrug resistance (MDR, resistant to ≥ 3 antimicrobial classes). The most common MDR phenotypes were AM-E-P-TET, which accounted for 15% of the 12 O157 strains. All of the examined non-O157 strains showed resistance to trimethoprim/sulfamethoxazole. The resistance patterns of all non-O157 strains to tested antibiotics were as follows: erythromycin (98%), penicillin (91%), ampicillin (73%), tetracycline (65%), chloramphenicol (40%), cefixime (25%) and enrofloxacin (21%). Forty-eight (92%) of the 52 non-O157 strains displayed multidrug resistance. The most frequently observed MDR profiles AM-C-E-P-TET-ENR–SXT (33% of the 52 non-O157 strains) were associated with 10 of these isolates (PNU4, PNU29, PNU31, PNU33, PNU38, PNU41, PNU49, PNU50, PNU51, PNU52). The resistance patterns of 64 STEC isolates are shown in Table 3 and Figure 3.

**TABLE 3:** Antibiotic resistance pattern in Shiga toxin–producing *Escherichia coli* strains.

STEC - Serogroup	No. positive		AM10		C30		CFM5		E25		ENR10		P10		SXT30		TET30	
	Sample	%	Sample	%	Sample	%	Sample	%	Sample	%	Sample	%	Sample	%	Sample	%	Sample	%
Non-O157	52	100.0	38	73.0	21	40.0	13	25.0	51	98.0	11	21.1	51	98.0	52	100.0	34	65.3
O157	12	18.7	10	83.0	0		3	25.0	8	66.6	0	-	11	91.6	0		8	66.6
Total STEC	64	100.0	48	75.0	21	32.8	16	25.0	59	92.1	11	17.1	62	96.8	25	39.0	42	65.6

AM10, ampicillin (10 *µ*g/disk); TET30, tetracycline (30 *µ*g/disk); E25, erythromycin (25 *µ*g/disk); ENR10, enrofloxacin (10 *µ*g/disk); SXT30, trimethoprim/sulfamethoxazole (30 *µ*g/disk); C30, chloramphenicol (30 *µ*g/disk); P10, penicillin (10 *µ*g/disk); CFM5, cefixime (5 *µ*g/disk); STEC, Shiga toxin-producing *Escherichia coli*; No., number.

### Integrons

**FIGURE 4:**
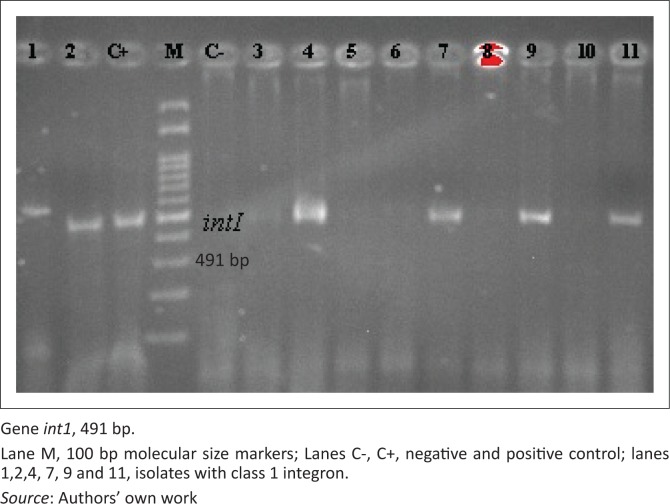
Polymerase chain reaction amplicons of Shiga toxin-producing *Escherichia coli* integrons. Polymerase chain reaction amplification of the class 1 integron, integrase. bp, base pair; *int1*, integrase gene.

Class 1 integrons were detected among 23 (36%) of the STEC isolates (Figure 4 and Table 4). Integron-positive strains were significantly more resistant to enrofloxacin, trimethoprim/sulfamethoxazole and tetracycline than integron-negative strains (*p* < 0.05). Nevertheless, resistance to ampicillin, erythromycin, penicillin, cefixime and chloramphenicol could not be directly related to the presence of integrons (Table 5). All of the integron-positive strains displayed multidrug resistance. The most prevalent MDR phenotypes in integron-positive strains were AM-CFM-E-P-TET (26% of the 52 non-O157 strains).

**TABLE 4:** Overview of the integron-positive Shiga toxin–producing *Escherichia coli* strains.

Strain	Serogroup	Virulence profile *stx1/2* gene	Integron *int* gene	Antibiotic resistance profile								MDR
				AM	C	CFM	E	ENR	P	SXT	TET	
PNU1	Non-157	1	+	R	S	S	R	S	R	R	R	+
PNU2	Non-157	2	+	S	S	S	R	S	R	R	R	+
PNU3	Non-157	2	+	R	R	S	R	R	S	R	R	+
PNU4	Non-157	2	+	R	R	S	R	R	R	R	R	+
PNU5	Non-157	2	+	R	S	S	R	S	R	S	R	+
PNU7	Non-157	1	+	R	S	R	R	S	R	S	R	+
PNU10	Non-157	1	+	R	S	R	R	S	R	S	R	+
PNU20	O157	1, 2	+	R	S	R	R	S	R	S	R	+
PNU21	O157	1, 2	+	R	S	R	R	S	R	S	R	+
PNU24	O157	1, 2	+	R	S	S	R	S	R	S	R	+
PNU26	O157	1, 2	+	S	S	S	R	S	R	S	S	-
PNU30	Non-157	2	+	R	R	S	R	R	R	R	R	+
PNU31	Non-157	2	+	R	R	S	R	R	R	R	R	+
PNU33	Non-157	1	+	R	R	S	R	R	R	R	R	+
PNU34	Non-157	2	+	S	R	S	R	S	R	R	R	+
PNU35	Non-157	2	+	R	S	R	R	S	R	S	R	+
PNU39	Non-157	2	+	S	R	S	R	S	R	R	R	+
PNU40	Non-157	1	+	R	S	S	R	R	R	R	R	+
PNU50	Non-157	1	+	R	R	S	R	R	R	R	R	+
PNU51	Non-157	2	+	R	R	R	R	S	R	R	R	+
PNU58	Non-157	2	+	R	S	S	R	S	R	R	R	+
PNU61	Non-157	2	+	R	R	S	R	S	R	R	R	+
PNU62	Non-157	2	+	R	S	S	R	S	R	R	R	+

Note: Antibiotic resistance profile was determined for eight antibiotics: ampicillin (AM), tetracycline (TET), erythromycin (E), enrofloxacin (ENR), trimethoprim/sulfamethoxazole (SXT), chloramphenicol (C), penicillin (P) and cefixime (CFM).

*stx*, isolates carrying *stx1* and/or *stx2* genes; MDR, multidrug-resistant isolates; S, antibiotic-susceptible isolates; R, antibiotic-resistant isolates.

+, positive for *int* gene or MDR.

**TABLE 5:** Comparison of the resistances between integron-positive and integron-negative strains was done using the *p*-values listed in the table.

Antibiotic	Resistance *int*-positive isolates		Resistance *int*-negative isolates		Resistance of total isolates		Association with integron
	No.	%	No.	%	No.	%	
Ampicillin	19	29.6	28	42.2	47	71.8	0.2523
Erythromycin	23	35.9	39	60.9	62	96.8	0.5322
Penicillin	22	34.3	40	62.5	62	96.8	1.0000
Tetracycline	21	32.8	20	29.6	41	62.5	0.0009*
Trimethoprim/sulfamethoxazole	15	23.4	10	15.6	15	39.00	0.0032*
Cefixime	7	10.9	9	14.1	16	25.00	0.6521
Enrofloxacin	7	10.9	4	6.2	11	17.1	0.0456*
Chloramphenicol	10	15.6	11	17.1	21	32.8	0.2780

Note: *p* values of 0.05 were considered to be significant.

*int*-positive, integron-positive in PCR assay; *int*-negative, integron-negative in PCR assay.

*int*, integron; No., number.

* , Correlation is significant at the 0.05 level.

## Discussion

Antibiotic resistance developed in STEC isolates from humans and animals (Van Meervenne et al. 2013). Integrons, which are known to be associated with many antimicrobial resistance genes, were suspected to serve as pools of antimicrobial resistance genes worldwide (El-Sokkary & Abdelmegeed 2015). Class 1 integrons are commonly found in gram-negative pathogens (Maguire et al. 2001).

In this study, the presence of major virulence factors and resistance to antimicrobials belonging to classes generally utilised in Iran was investigated in zoonotic STEC isolates from calves with diarrhoea. Owing to the close contact of humans with animals, the presence of virulence and antimicrobial resistance genes in *E. coli* strains harboured by animals leads to public health concerns (Torkan et al. 2016). *Escherichia coli,* which has been implicated as an aetiological factor of calf diarrhoea, harbours many virulence genes that enable it to cause disease in a particular host (Nagarjuna et al. 2015). In the present study, among 312 *E. coli* strains from diarrhoeic calves, 71 (22.7%) were STEC. The results are in agreement with those of Dastmalchi et al. (2012), who screened 51 *E. coli* isolates from diarrhoeic calves in the Urmia region, which is located in west Azerbaijan Province, Iran, and illustrated that 19.6% of isolates were *stx* positive. Most epidemiological studies in diarrhoeic calves in Iran have disclosed that the prevalence of STEC infection ranges between 6.4% and 34.5% (Pourtaghi, Dahpahlavan & Momtaz 2013; Shahrani et al. 2014). These discrepancies can be attributed to the small sample size and geographical differences. In other words, STEC prevalence in calves may be influenced by environmental factors (Dastmalchi et al. 2012). Higher prevalence of the *stx2* gene (54 isolates) compared to the *stx1* gene (33 isolates) in this study corroborates the findings of previous reports in Iran (Dastmalchi et al. 2012; Tahamtan, Hayati & Namavari 2010). However, these results contrast with other reports that have shown that most STEC from diarrhoeic calves only produce *stx1*, whereas *stx2*-positive strains are the dominant types in healthy calves (Nguyen, Vo & Vu-Khac 2011). The differences in these findings suggest that *stx2* may be associated with a majority of *E. coli* isolates from diarrhoeic calves in Iran. Shiga toxin producing *E. coli* infection, which is associated with diarrhoea in calves, may result in severe diseases in humans such as HUS and HC (Bastos et al. 2006). The diarrhoeal phase of diseases associated with STEC is usually self-limiting, and the role of early antimicrobial treatment in the prevention of HUS is still regarded as controversial (Shahrani et al. 2014). Current recommendations and the available data suggest that not only do antibiotic exposure increase the risk of HUS in children via inducing expression of *stx* through replication of temperate *bacteriophages* carrying *stx*-encoding *genes* (Ochoa et al. 2007), it turns out to have another perilous effect on the frequency of STEC antimicrobial resistance (Shahrani et al. 2014), which could result in an increase of frequency of STEC and perhaps greater shedding. Resistance could contribute to *g*reater contamination of animal food products with STEC (Torkan et al. 2016). Several reports have documented that a significant increase of antimicrobial resistance in STEC strains isolated from animals and humans has acquired antibiotic resistance genes almost 20 years ago (Zhao et al. 2001). In STEC strains, class 1 integrons are strongly associated with multidrug resistance (Colello et al. 2015). Previous studies have reported the occurrence and prevalence of class 1 integrons to be ranging from 2.7% to 41.0% among STEC isolates in Germany (Askar et al. 2011), Argentina (Colello et al. 2015), Belgium (Van Meervenne et al. 2013), North America (Nagachinta & Chen 2009), Brazil (Cergole-Novella et al. 2011) and US (Singh et al. 2005; Zhao et al. 2001). Class 1 integrons appear to be common in the endemic STEC strains. In the present study, class 1 integron was identified in 23 (36%) out of 71 STEC isolates. Our data revealed low distribution of class 1 integrons among STEC isolates from calves with diarrhoea in the south of Iran compared with a similar study in northern Iran in 2014 for which the authors found a higher percentage (53%) of the strains containing integron class 1 (Bakhshi et al. 2014). The various percentages of class 1 integrons in different parts of the world could be attributed to the characteristics of the analysed collection and differences in the prevalence of antibiotic consumption in each country (Kargar et al. 2014). In general, exposure to antibiotics, heavy metals or biocides and a high multiplicity of other different environmental factors are among the main reasons for an increase of cells containing integrons (Baquero, Martínez & Cantón 2008).

All integron-positive strains examined in this study were resistant to at least three different antibiotics (MDR). Similarly, high percentages of MDR phenotypes among integron-positive STEC strains have been reported in Argentina (Nagachinta & Chen 2009) and Iran (Bakhshi, Najibi & Sepehri-Seresht 2014). However, other authors (Colello et al. 2015; Van Meervenne et al. 2013) have reported a lower rate (less than 90%) of STEC in diarrhoeic calves. The highest resistances among the integron-positive strains were found to enrofloxacin (17%), trimethoprim/sulfamethoxazole (39%) and tetracycline (62%). The integron-positive strains were significantly more resistant to these antibiotics than the integron-negative strains. The resistance to enrofloxacin (ENR), trimethoprim/sulfamethoxazole (SXT) and tetracycline (TET) is related to the presence of the integron. The significant association between resistance to fluoroquinolones, tetracycline, trimethoprim and sulfonamides (ENR, TET, SXT) tested and integron existence could be explained because of the fact that many fluoroquinolone, tetracycline, trimethoprim and sulfonamide resistant genes have been reported within integron structures, including *gyrA, gyrB, qnr, tetA, tetB, tetC, tetD, sul1, sul2, sul3* and *dfrA1* (Kaplan et al. 2013; Wang et al. 2010).

## Conclusion

We report the presence of class 1 integrons in the most familiar STEC strains from diarrhoeic calves. Results imply that *stx2, stx1* and *eae* putative virulence gene, the *IntI* integrase gene and resistance to erythromycin, penicillin, ampicillin, tetracycline and trimethoprim/sulfamethoxazole were the most commonly detected characteristics of the STEC strains isolated from diarrhoeic calves in southern Iran. Our investigation demonstrated that calves are possible reservoirs of STEC strains and developed resistance to multiple classes of antimicrobials. Emerging data suggest an association between MDR and integrons which may play a significant role in the dissemination of resistance genes. Therefore, it is advised to stop routine antimicrobial treatment and conduct further molecular studies to detect other antimicrobial resistance and virulent genes in STEC isolates obtained in this study.
